# The quality of life impacting factors in malnourished patients with gastric cancer

**DOI:** 10.3389/fonc.2024.1336859

**Published:** 2024-04-25

**Authors:** Hong Zhao, Chenan Liu, Guotian Ruan, Xin Zheng, Yue Chen, Shiqi Lin, Xiaoyue Liu, Jinyu Shi, Xiangrui Li, Shuqun Li, Hanping Shi

**Affiliations:** ^1^ Department of Gastrointestinal Surgery/Department of Clinical Nutrition, Beijing Shijitan Hospital, Capital Medical University, Beijing, China; ^2^ Key Laboratory of Cancer FSMP for State Market Regulation, Beijing, China; ^3^ National Clinical Research Center for Geriatric Diseases, Xuanwu Hospital, Capital Medical University, Beijing, China

**Keywords:** gastric cancer, quality of life, malnourishment, PG-SGA, young and old

## Abstract

**Introduction:**

Malnutrition is prevalent among individuals with gastric cancer and notably decreases their quality of life (QOL). However, the factors impacting QOL are yet to be clearly defined. This study aimed to identify essential factors impacting QOL in malnourished patients suffering from gastric cancer.

**Methods:**

By using the Patient-Generated Subjective Global Assessment (PG-SGA) to assess the nutritional status (≥4 defined malnutrition) of hospitalized cancer patients, 4,586 gastric cancer patients were ultimately defined as malnourished. Spearman method was used to calculate the relationship between clinical features and the European Organization for Research and Treatment of Cancer Quality of Life Questionnaire (EORTC QLQ-C30). Then, univariate and multivariate logistic regression were used to observe which factors affected QOL, and subgroup analysis was performed in young and old population respectively. In addition, we used univariate and multivariate logistic regression to explore whether and how self-reported frequent symptoms in the last 2 weeks of the PG-SGA score affected QOL.

**Results:**

In multivariate logistic regression analysis of clinical features of patients with malnourished gastric cancer, women, stage II, stage IV, WL had an independent correlation with a low global QOL scores. However, BMI, secondary education, higher education, surgery, chemotherapy, HGS had an independent correlation with a high global QOL scores. In multivariate logistic regression analysis of symptoms in self-reported PG-SGA scores in patients with malnourished gastric cancer, having no problem eating had an independent correlation with a high global QOL scores. However, they have no appetite, nausea, vomiting, constipation and pain had an independent correlation with a lower global QOL scores. The p values of the above statistical results are both < 0.05.

**Conclusion:**

This study demonstrates that QOL in malnourished patients with gastric cancer is determined by female sex, stage II, stage IV, BMI, secondary and higher education or above, surgery, chemotherapy, WL, and HGS. Patients’ self-reported symptoms of nearly 2 weeks, obtained by using PG-SGA, are also further predictive of malnourished gastric cancer patients. Detecting preliminary indicators of low QOL could aid in identifying patients who might benefit from an early referral to palliative care and assisted nursing.

## Introduction

Despite variations in incidence and mortality rates across different regions, gastric cancer is the fifth most commonly detected cancer worldwide and the fourth most common cause of death due to cancer (1). In China, it is the second leading cause of death related to cancer (2). The incidence and progression of this disease is determined by an interplay of environmental and genetic factors, indicating that gastric cancer is multifactorial in nature (3). Currently, the management of gastric cancer is far from optimal, given that patients, irrespective of their disease subtype, generally receive uniform treatment (4). Recently, there has been a shift in discussion and decision-making about cancer care, especially when considering patient selection, from a variety of clinical outcomes to patient-centered outcomes such as QOL (5). There has also been a significant evolution in palliative care and treatment approaches, where the objective is to unify life-extending treatments with patient QOL (4, 6–11). Although new therapies and technologies can improve treatment outcomes in cancer patients, it is of equal importance to maintain physical and emotional health by assessing QOL (12, 13), which is negatively affected by cancer (14). Patients with late-stage or uncontrollable gastric cancer constantly experience malnutrition, which affects their QOL, increases the chemotherapy toxicity and reduces the overall survival rate (15, 16). Despite the widespread prevalence of gastric cancer worldwide, our knowledge about its effect on QOL is still limited (17). The goal of this research is to assess the factors impacting the QOL of malnourished patients with gastric cancer. The findings of this study will enhance care strategies and management of patients, and offer vital references for future clinical practice and research.

## Materials and methods

### Study population

The INSCOC is a nationwide survey exploring the link between nutritional health and clinical results in patients suffering from malignant tumors. This project was both conceived and put into action by the Tumor Nutrition and Support Professional Committee within the Chinese Cancer Society. Ethical approval for the study was granted by the reviewing bodies of all participating institutions, with all participants giving their informed written consent. The criteria for participation in this study were as follows:

1. Individuals aged 18 to 90 years with full mental capacity, no communication issues, and capable of participating in the necessary examinations.
2. Histological diagnosis of gastric cancer.
3. Experienced multiple hospitalizations for the same condition.
4. Comprehensive documentation of medical history and any subsequent data.
5. Able to voluntarily participate.

The exclusion criteria were as follows:

1. Patients with HIV/AIDS or organ transplant recipients.
2. Patients in critical condition or difficult to evaluate.
3. Patients who refuse or do not cooperate with the survey questionnaire.

### Procedure and assessment

We use Investigation on Nutrition Status and Clinical Outcome of Common Cancers (INSCOC) data screened 5,845 eligible adult patients with gastric cancer from 26 provinces and municipalities in China between 2012 and 2022. Professional staff used a standardized questionnaire and professional measurement methods to collect information on sex, age, TNM stage, body mass index (BMI) (18), the rate of weight loss over one month (WL), hand grip strength (HGS),occupation, education level, residence, and treatment within the initial 48 hours of hospital admission. Missing data is interpolated using R software (**Supplementary Figure 1**). By Asian standards, HGS is classified as low grip strength (HGS<18 for Female; HGS<28 for Male) and high grip strength (HGS≥18 for Female; HGS≥28 for Male) (19). Occupations are divided into mental work, manual work and retired or other. Mental work includes professional or managerial personnel, civil servants, teachers, career and enterprise staff. Manual work includes farmers and workers. The level of education is divided into higher education (college, bachelor’s, master’s and above), secondary education (middle and high school) or no education and primary education. Patient-Generated Subjective Global Assessment (PG-SGA) and the European Organization for Research and Treatment of Cancer Quality of Life Questionnaire (EORTC QLQ-C30) (20) are obtained by the investigators in the form of filling in questionnaires after the patients understand the questions and explain the questions. In this way, the errors caused by the patients’ unclear understanding can be avoided to the maximum extent. The PG-SGA score is recognized by the American Society for Parenteral and Enteral Nutrition (ASPEN) as the benchmark nutritional assessment tool for cancer patients. The PG-SGA score is composed of patient self-assessment and a comprehensive assessment by the health care provider and is not a short form of nutritional risk screening. The PGSGA score is 0-1 (no intervention is required at this time, and routine follow-up and evaluation are maintained during treatment). The PG-SGA score is 2-3 (patient or patient family education by a dietitian, nurse, or physician, and medical intervention may be performed based on the presence of symptoms and the results of laboratory tests). The PG-SGA score of 4-8 (intervention by a dietitian and, depending on the severity of symptoms, in conjunction with a physician and caregiver). The PG-SGA score≥9 (urgent need for symptom improvement and/or concurrent nutritional intervention). In this study, PG-SGA≥4 was defined as malnutrition, and the higher the score, the more severe the malnutrition, on the contrary, the lower the score, the better the nutritional status. QOL was assessed using the EORTC QLQC30, which evaluates 5 functional scores (physical function, role function, emotional function, congnitive function, social function), global health, 3 symptom scales (fatigue, nausea and vomiting, pain), 6 individual measures (dyspnea, sleep disturbance, appetite loss, constipation, diarrhea, financial difficulties). The computation of each domain’s summary QOL score (0-100) adhered to the EORTC QLQ-C30 formulas. Higher scores on the functional and global health status scales indicate improved functioning. Conversely, Symptom scales and individual measurement items use negative scores, with higher scores indicating greater intensity. The total score of the QOL is added by each functional score, then added to 800 minus 3 symptom scores and 5 individual measures (except financial difficulties), and finally 13 (21).

### Statistical analysis

We employed a multiple imputation chain-equation method to impute missing data, assuming that the missingness was random. Predictive mean matching was used to impute missing continuous variables, while a logistic regression model was used for imputing missing binary variables. Through 100 iterations, we generated five imputed datasets and analyzed each separately. Finally, we combined the results using Rubin’s method. The data is presented as a simple percentage or median interquartile range (IQR). The correlation between clinical features of patients with malnourished gastric cancer and EORTC QLQ-C30 was calculated by the spearman method. The greater the absolute value of the correlation coefficient calculated by the spearman method, the stronger the correlation was. Then the OR is calculated by univariable and multivariable logistic regression. The OR>1 indicates that it is a predictor of outcome events (low global QOL scores, low physical function scores, high fatigue scores, high appetite loss scores). The OR<1 indicates that it is a predictor of outcome events (high global QOL scores, high physical function scores, low fatigue scores, low appetite loss scores). The above scores are compared with the average. If in univariate and multivariate logistic regression, p values are both <0.05, indicating that this factor can independently affect outcome events. R software, version 4.3.0, was used for all analytical procedures.

## Results

### Baseline characteristics

The baseline characteristics of 5845 gastric cancer patients were shown in supplementary **Table 1**, of which 4586 (78.5%) were malnourished. In **Table 1**, we observed the baseline characteristics of 4586 malnourished patients with gastric cancer, 3149 men (68.7%) and 1437 women (31.3%). the median age was 60 years old, IQR (52.00, 67.00). Of the total number of patients, 546 patients in stage I (11.9%); 884 patients in stage II (19.3%); 1813 patients in stage III (39.5%), and 1,343 patients in stage IV (29.3%). The median BMI was 20.57 (IQR 18.44, 22.96). Less than half of patients were treated with surgery, chemotherapy or radiotherapy. More than half of the patients with gastric cancer experienced WL (69.1%). There were 2358 (51.4%) people with low HGS and 2228 (48.6%) people with high HGS. 1720 (37.5%) of the study population had primary education or no education, 2259 (49.3%) had secondary education, and 607 (13.2%) had higher education. The proportion of patients living in urban and rural areas is similar.

**Table 1:** Characteristics of patients with malnourished gastric cancer.

Characteristics	Sample size (n=4586)
Sex	
Male	3149 ( 68.7)
Female	1437 ( 31.3)
Age	60.00 [52.00, 67.00]
TNM stage	
I	546 ( 11.9)
II	884 ( 19.3)
III	1813 ( 39.5)
IV	1343 ( 29.3)
BMI	20.57 [18.44, 22.96]
Surgery	
No	2639 ( 57.5)
Yes	1947 ( 42.5)
Chemotherapy	
No	2554 ( 55.7)
Yes	2032 ( 44.3)
Radiotherapy	
No	4477 ( 97.6)
Yes	109 ( 2.4)
WL%	
≤0	1418 ( 30.9)
0∼5	1383 ( 30.2)
5∼10	1211 ( 26.4)
>10	574 ( 12.5)
HGS	
<18 for women or <28 for men	2358 ( 51.4)
≥18 for women or≥28 for men	2228 ( 48.6)
Education	
Primary education or never attended school	1720 ( 37.5)
Secondary education	2259 ( 49.3)
Higher education	607 ( 13.2)
Occupation	
Mental work	495 ( 10.8)
Manual work	1896 ( 41.3)
Retired or other	2195 ( 47.9)
Residence	
Urban	2188 ( 47.7)
Rural	2398 ( 52.3)

The summary statistics present N% for categorical variables and median [IQR] deviation for continuous variables.

### Relationship between clinical and nutritional determinants and EORTC QLQC30 scores

Female (ρ-0.11), age (ρ-0.05), tumor stage (ρ-0.08) and WL (ρ-0.1) were negatively correlated with the global QOL score. BMI (ρ0.16), surgery (ρ0.04), chemotherapy (ρ0.07), HGS (ρ0.24) and education (ρ0.06) were positively correlated with the global score of QOL (**Tables 2**, **3**).

**Table 2:** Relationship between clinical and nutritional determinants and EORTC QLQC30 scores.

Varible	No.of patients	Physical function		Role function		Emotional function		Cognitive function		Social function		Global health		Global QOL	
Sex		ρ	P	ρ	P	ρ	P	ρ	P	ρ	P	ρ	P	ρ	P
Men, Women	4586	-0.1	<0.001	-0.08	<0.001	-0.08	<0.001	-0.06	<0.001	-0.06	<0.001	-0.07	<0.001	-0.11	<0.001
Age															
60.00 [52.00, 67.00]	4586	-0.12	<0.001	-0.05	<0.001	0.07	<0.001	-0.09	<0.001	-0.01	0.185	-0.04	0.001	-0.05	<0.001
Tumor stage															
I, II, III, IV	4586	-0.1	<0.001	-0.12	<0.001	-0.03	0.21	-0.04	0.033	-0.06	<0.001	-0.06	<0.001	-0.08	<0.001
BMI															
20.57 [18.44, 22.96]	4586	0.19	<0.001	0.14	<0.001	0.06	<0.001	0.1	<0.001	0.12	<0.001	0.15	<0.001	0.16	<0.001
Surgery															
No or Yes	4586	0.13	<0.001	0.14	<0.001	-0.04	0.001	0.06	0.004	0.08	<0.001	0.01	0.731	0.04	0.015
Chemotherapy															
No or Yes	4586	0	<0.001	-0.04	0.602	0.08	<0.001	0.03	0.002	0	0.247	0.08	<0.001	0.07	<0.001
Radiotherapy															
No or Yes	4586	-0.01	0.69	-0.01	0.888	0	0.736	0	0.596	-0.01	0.358	-0.02	0.213	-0.02	0.549
WL															
≤0%, 0-5%, 5%-10%, >10%	4586	-0.08	<0.001	-0.08	<0.001	-0.04	<0.001	-0.02	0.091	-0.05	<0.001	-0.08	<0.001	-0.1	<0.001
HGS															
<18 for women or <28 for men, ≥18 for women or≥28 for men	4586	0.28	<0.001	0.2	<0.001	0.09	<0.001	0.18	<0.001	0.19	<0.001	0.19	<0.001	0.24	<0.001
Education															
Primary education or never attended school, Secondary education, Higher education	4586	0.06	0.014	0.02	0.657	0.03	0.076	0.1	<0.001	0.07	<0.001	0.07	<0.001	0.06	0.004
Occupation															
Mental work, Manual work, Retired or other	4586	-0.07	<0.001	-0.07	<0.001	0.04	0.028	-0.02	0.018	0.02	0.165	-0.01	0.649	-0.01	0.359
Residence															
Urban, Rural	4586	0.04	0.001	0.04	0.001	-0.04	0.066	0	0.716	-0.03	0.032	-0.07	<0.001	-0.01	0.339

ρ, correlation coefficient.

**Table 3:** Relation Between Clinical and Nutritiona Parameters With European Organization for Research and Treatment of Cancer Quality-of-Life Symptom Scales.

Varible	No.of patients	Fatigue		Nausea and vomiting		Pain		Dyspnea		Sleep disturbance		Appetite loss		Constipation		Diarrhea		Financial difficulties	
Sex		ρ	P	ρ	P	ρ	P	ρ	P	ρ	P	ρ	P	ρ	P	ρ	P	ρ	P
Men, Women	4586	0.08	<0.001	0.09	<0.001	0.06	<0.001	0.02	0.108	0.09	<0.001	0.09	<0.001	0.01	0.308	0.04	0.001	0.03	0.014
Age																			
60.00 [52.00, 67.00]	4586	0.06	<0.001	-0.03	0.205	-0.04	0.161	0.07	<0.001	-0.01	0.628	0.05	<0.001	0.04	0.018	-0.02	0.578	-0.06	0.002
Tumor stage																			
I, II, III, IV	4586	0.08	<0.001	0.09	<0.001	0.02	0.165	0.03	0.027	0.03	0.225	0.08	<0.001	0.06	<0.001	0	0.678	-0.01	0.188
BMI																			
20.57 [18.44, 22.96]	4586	-0.16	<0.001	-0.1	<0.001	-0.03	0.037	-0.06	0.001	-0.1	<0.001	-0.1	<0.001	-0.09	<0.001	-0.05	<0.001	-0.1	<0.001
Surgery																			
No or Yes	4586	-0.06	0.001	-0.01	0.334	0.15	<0.001	-0.02	0.217	-0.01	0.803	-0.04	0.118	-0.04	0.008	-0.03	0.043	0.03	0.005
Chemotherapy																			
No or Yes	4586	-0.03	<0.001	-0.04	0.001	-0.2	<0.001	-0.04	0.002	-0.05	<0.001	-0.04	<0.001	-0.03	0.012	0.01	0.819	-0.07	<0.001
Radiotherapy																			
No or Yes	4586	0	0.71	0.01	0.279	0	0.62	0.02	0.267	0.01	0.683	0.03	0.109	0.02	0.132	0.01	0.545	0.03	0.085
WL																			
≤0%, 0-5%, 5%-10%, >10%	4586	0.11	<0.001	0.07	<0.001	0.02	0.019	0.03	0.027	0.07	<0.001	0.08	<0.001	0.07	<0.001	0.03	0.029	0.04	0.006
HGS																			
<18 for women or <28 for men, ≥18 for women or≥28 for men	4586	-0.22	<0.001	-0.08	<0.001	-0.09	<0.001	-0.12	<0.001	-0.11	<0.001	-0.13	<0.001	-0.08	<0.001	-0.06	<0.001	-0.12	<0.001
Education																			
Primary education or never attended school, Secondary education, Higher education	4586	-0.02	0.718	-0.03	0.181	-0.04	0.095	-0.01	0.574	-0.04	0.035	-0.04	0.01	0	0.954	0.01	0.387	-0.17	<0.001
Occupation																			
Mental work, Manual work, Retired or other	4586	-0.01	0.627	-0.02	0.427	-0.03	0.147	0.01	0.171	-0.01	0.922	-0.01	0.688	0.02	0.077	0.01	0.183	-0.09	<0.001
Residence																			
Urban, Rural	4586	-0.01	0.499	0	0.738	0.05	0.032	-0.03	0.017	0.02	0.683	0.01	0.702	-0.02	0.045	-0.05	0.001	0.17	<0.001

ρ, correlation coefficient.

### Clinical and nutritional determinants related to poorer global QOL scores

**Figure 1:**
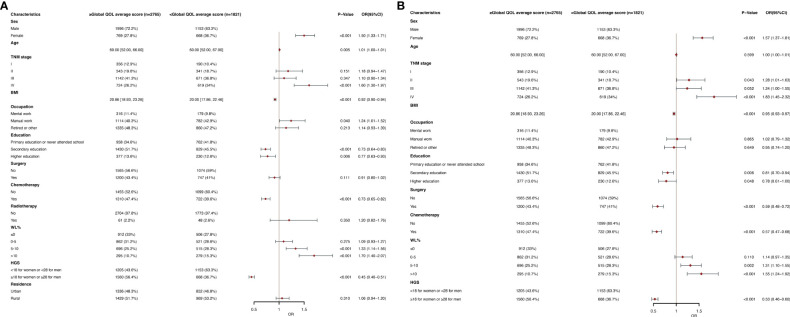
**(A)** Clinical and Nutritional Parameters Related to Poor Global QOL average score (Below the Mean of <82.83) According to Univariable Logistic Regression Analysis. OR, odds ratio; CI, confidence interval; The summary statistics present N% for categorical variables and median [IQR] deviation for continuous variables. **(B)** Clinical and Nutritional Parameters Related to Poor Global QOL average score (Below the Mean of <82.83) According to Multivariable Logistic Regression Analysis. OR, odds ratio; CI, confidence interval; The summary statistics present N% for categorical variables and median [IQR] deviation for continuous variables. .

The global QOL scores was segmented based on the mean score (82.83) in the univariable and multivariate logistic regression analysis (**Figures 1A, B**). Female sex (OR, 1.57; 95% CI, 1.37-1.81; P<0.001), stage II (OR, 1.28; 95% CI, 1.01-1.63; P =0.043), stage IV (OR, 1.83; 95% CI, 1.45-2.32; P<0.001) and WL (WL 5-10%: OR, 1.31; 95% CI, 1.10-1.55; P =0.002; WL>10%: OR, 1.55; 95% CI, 1.24-1.92; P<0.001) were an independent predictor of a lower global QOL scores (<82.83). BMI (OR, 0.95; 95% CI, 0.93-0.97; P<0.001), secondary education (OR, 0.81; 95% CI, 0.70-0.94; P =0.006), higher education (OR, 0.78; 95% CI, 0.61-1.00; P =0.048), surgery (OR, 0.59; CI, 0.48-0.72; P < 0.001), chemotherapy (OR, 0.57; 95% CI, 0.47-0.68;P<0.001) and high HGS (OR, 0.53; 95% CI, 0.46-0.60; P< 0.001) were an independent predictor of higher global QOL scores (≥82.83). The study population was divided into young group (<65) and elderly group (≥65), and subgroup analysis was performed. We found that in the elderly population, the age (OR, 1.03; 95% CI, 1.01-1.05; P =0.008) was an independent predictor of low global QOL scores, and other results were similar to those of the total population analysis (**Supplementary Table 3B**). In the young age group, the results obtained are similar to the results of the general population analysis (**Supplementary Table 3A**).

### Parameters related to clinical and nutritional aspects linked to diminished physical function

**Figure 2:**
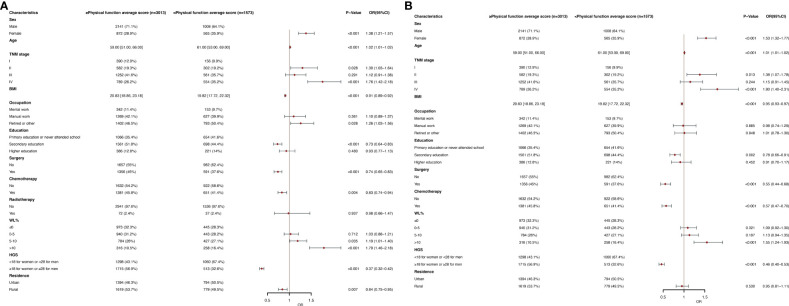
**(A)** Clinical and Nutritional Parameters Related to Poor Physical function average score (Below the Mean of <78.67) According to Univariable Logistic Regression Analysis. OR, odds ratio; CI, confidence interval; The summary statistics present N% for categorical variables and median [IQR] deviation for continuous variables. **(B)** Clinical and Nutritional Parameters Related to Poor Physical function average score (Below the Mean of <78.67) According to Multivariable Logistic Regression Analysis. OR, odds ratio; CI, confidence interval; The summary statistics present N% for categorical variables and median [IQR] deviation for continuous variables.

**Figures 2A, B** show the physical function in The EORTC QLQC30 score divided by an average score (78.67). In multivariate logistic regression analysis, women (OR, 1.53; 95% CI, 1.32-1.77; P< 0.001), age (OR, 1.01; 95% CI, 1.01-1.02; P <0.001), stage II (OR, 1.38; 95% CI, 1.07-1.78; P =0.013), stage IV (OR, 1.80; 95% CI, 1.40-2.31; P <0.001) and WL >10 (OR, 1.55; 95% CI, 1.24-1.93; P <0.001) were associated with poor physical function (< 78.67) of independent predictors. BMI (OR, 0.95; 95% CI, 0.93-0.97; P <0.001), secondary school (OR, 0.78; 95% CI, 0.66-0.91; p=0.002), surgery (OR, 0.55; 95% CI, 0.44-0.68; p<0.001), chemotherapy (OR, 0.57; 95% CI, 0.47-0.70; p<0.001) and high HGS(OR, 0.46; 95% CI, 0.40-0.53; p<0.001) were an independent predictor of better physical function (≥78.67). In a subgroup analysis of age, in the younger age group, results were obtained that were similar to the general population (**Supplementary Table 4A**). In the elderly population, the age (OR, 1.06; 95% CI, 1.04-1.09; p<0.001) became an independent predictor of lower physical function scores (**Supplementary Table 4B**).

### Parameters related to clinical and nutritional aspects linked to increased fatigue

**Figure 3:**
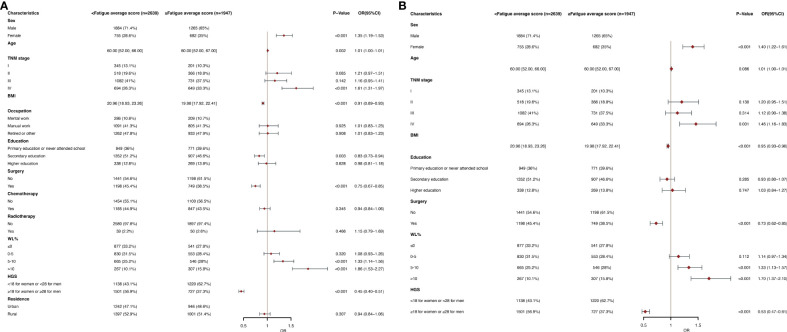
**(A)** Clinical and Nutritional Parameters Related to Increased Fatigue average score (Above the Mean of >24.15) According to Univariable Logistic Regression Analysis. OR, odds ratio; CI, confidence interval; The summary statistics present N% for categorical variables and median [IQR] deviation for continuous variables. **(B)** Clinical and Nutritional Parameters Related to Increased Fatigue average score (Above the Mean of >24.15) According to Multivariable Logistic Regression Analysis. OR, odds ratio; CI, confidence interval; The summary statistics present N% for categorical variables and median [IQR] deviation for continuous variables.

**Figures 3A, B** show the fatigue in The EORTC QLQC30 score divided by an average score (24.15). In multivariate logistic regression analysis, women (OR, 1.40; 95% CI, 1.22-1.61; p<0.001), stage IV (OR, 1.46; 95% CI, 1.16-1.83; P =0.001) and WL of 5-10 (OR, 1.33; 95% CI, 1.13-1.57; p<0.001), WL of >10 (OR, 1.70; 95% CI, 1.37-2.10; p<0.001) were an independent predictor of higher fatigue scores (≥24.15). BMI (OR, 0.95; 95% CI, 0.93-0.96; p<0.001), surgery (OR, 0.73; 95% CI, 0.62-0.85; p<0.001), and high HGS (OR, 0.53; 95% CI, 0.47-0.61; p<0.001) were a low fatigue scores (<24.15) of independent predictors. In subgroup analysis, in the elderly population, the age (OR, 1.03; 95% CI, 1.01-1.05; p=0.007) was an independent predictor of higher fatigue scores, and other observations were similar (**Supplementary Table 5B**). Results were observed in younger age groups similar to the general population (**Supplementary Table 5A**).

### Parameters related to clinical and nutritional aspects linked to increased appetite loss

**Figure 4:**
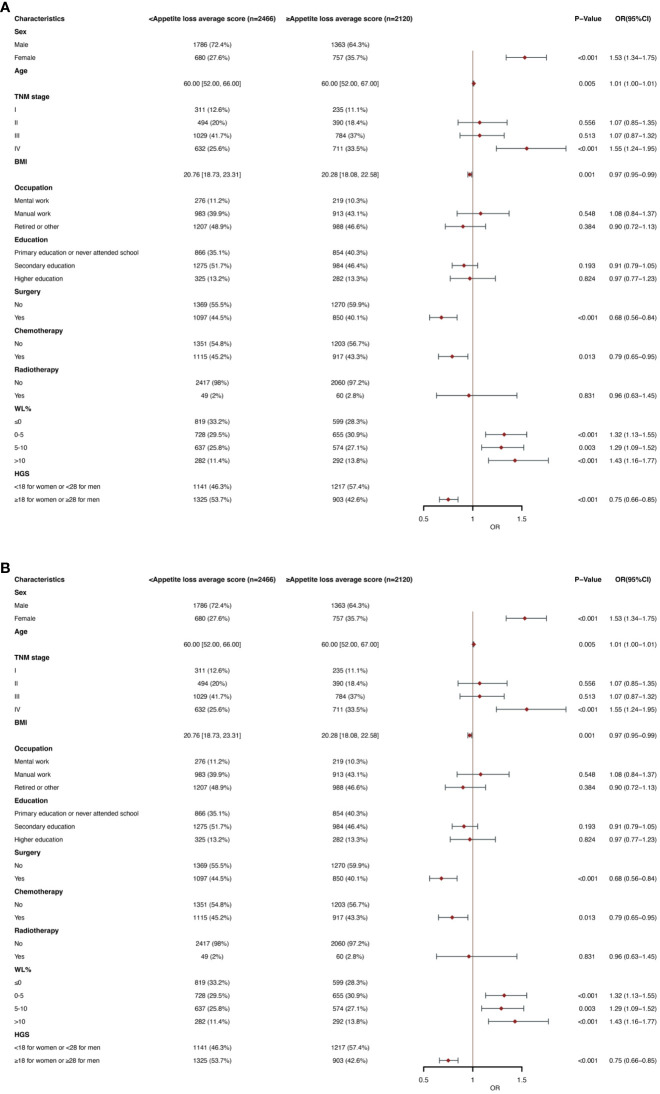
**(A)** Clinical and Nutritional Parameters Related to Increased Appetite loss average score (Above the Mean of >21.56) According to Univariable Logistic Regression Analysis. OR, odds ratio; CI, confidence interval; The summary statistics present N% for categorical variables and median [IQR] deviation for continuous variables. **(B)** Clinical and Nutritional Parameters Related to Increased Appetite loss average score (Above the Mean of >21.56) According to Multivariable Logistic Regression Analysis. OR, odds ratio; CI, confidence interval; The summary statistics present N% for categorical variables and median [IQR] deviation for continuous variables.

**Figures 4A, B** show that appetite loss in the EORTC QLQC30 score was divided by mean score (21.56). In multivariate logistic regression analysis, female (OR, 1.53; 95% CI, 1.34-1.75; p< 0.001), age (OR, 1.01; 95% CI, 1.00-1.01; p=0.005), stage IV (OR, 1.55; 95% CI, 1.24-1.95; P < 0.001), WL of 0-5 (OR, 1.32; 95% CI, 1.13-1.55; P<0.001), WL of 5-10 (OR, 1.29; 95% CI, 1.09-1.52; P =0.003) and WL >10 (OR, 1.43; 95% CI, 1.16-1.77; P < 0.001) were an independent predictor of higher appetite loss scores (≥21.56). BMI (OR, 0.97; 95% CI, 0.95-0.99; P =0.001), surgery (OR, 0.68; 95% CI, 0.56-0.84; P< 0.001), chemotherapy (OR, 0.79; 95% CI, 0.65-0.95; P =0.013) and high HGS (OR, 0.75; 95% CI, 0.66-0.85; P <0.001) were a lower appetite loss scores (< 21.56) of independent predictors. In the subgroup analysis of age, similar results were obtained in the older and younger groups, respectively (**Supplementary Tables 6A, B**).

### The relationship between PG-SGA symptoms and the global QOL scores

**Figure 5:**
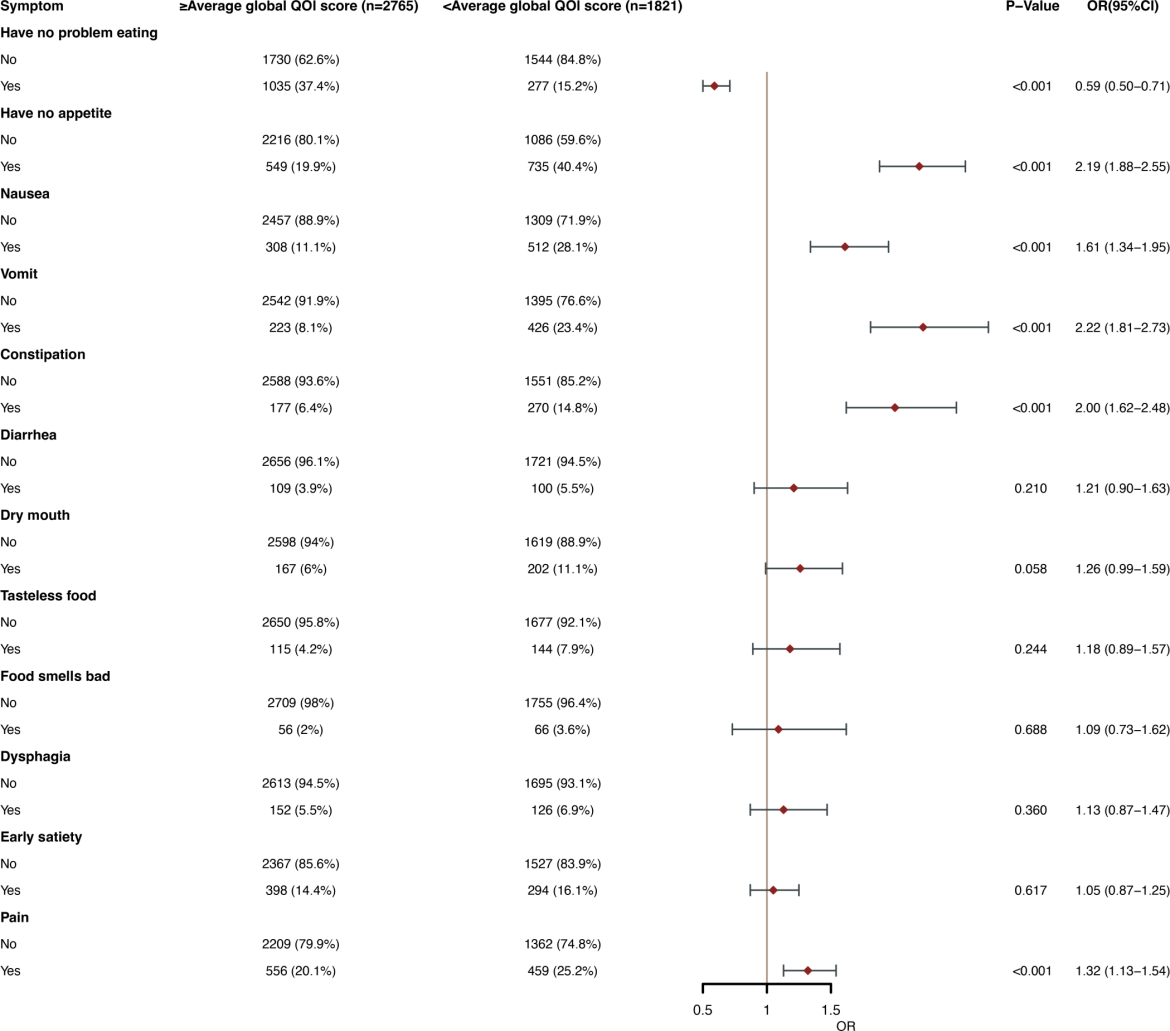
Multivariate logistic regression between patients' self-reported PG-SGA symptoms and poor global QOL average score (<82.83) in the past 2 weeks. OR, odds ratio; CI, confidence interval; The summary statistics present N% for categorical variables and median [IQR] deviation for continuous variables.

As part of the added value of PG-SGA, we also explored the relationship between self-reported symptoms and global QOL scores in the last 2 weeks. In multivariate logistic regression analysis, having no problem eating (OR, 0.59; 95% CI, 0.50-0.71; P < 0.001) was an independent predictor of high QOL scores. Having no appetite (OR, 2.19; 95% CI, 1.88-2.55; p< 0.001), nausea (OR, 1.61; 95% CI, 1.34-1.95; P<0.001), vomiting (OR, 2.22; 95% CI, 1.81-2.73; p<0.001), constipation (OR, 2.00; 95% CI, 1.62-2.48; P <0.001) and pain (OR, 1.32; 95% CI, 1.13-1.54; P <0.001) were an independent predictor of low QOL scores (**Figure 5**).

## Discussion

Our results suggest that women are independent predictors of lower global QOL scores, lower physical function scores, higher fatigue scores, and higher appetite loss scores, and that more attention should be paid to older patients when it comes to QOL in cancer patients. The symptoms of PG-SGA that occurred frequently in the last 2 weeks were also correlated with QOL scores.

Globally, gastric cancer continues to be a major contributor to cancer-related deaths, owing to its high mortality rate. This is largely because most diagnoses occur at later stages, when prognosis is often poor and treatment options are limited (22, 23). Alleviating symptoms, particularly malnourishment, and improving QOL should be a main objective of care (24). As far as we know, this is the first study to report an extensive examination of how the clinical aspects of nutrition impact the QOL of malnourished patients diagnosed with gastric cancer. Our results indicate that female patients may experience malnutrition more often than men. This is consistent with earlier studies, which also show a heightened risk of malnutrition in women with cancer (25). Therefore, when formulating nutritional assistance programs, it is crucial to focus on the dietary requirements of female patients.

Malnutrition poses a significant risk to patients with cancer as their nutritional condition can be compromised by both the illness itself and its treatment (26). Understanding the epidemiology of malnutrition could aid in the early management of complications during treatment, potentially improving patient QOL, the intensity of treatment, and outcome (27). Therefore, healthcare professionals should assess the nutritional condition of patients with gastric cancer and offer appropriate interventions or treatments for those suffering from malnutrition.

Individuals diagnosed with stage 4 gastric cancer were more prone to malnutrition, potentially due to interference from the tumor, which obstructs the normal functioning of the pylorus or duodenum and leads to inadequate intake. These patients also experience a surge in metabolic demands, which deteriorates their QOL and physical capacity (28). The results of our analysis are consistent with those reported in previous studies where patients with advanced or uncontrollable stomach cancer often suffer from malnutrition, which can impact their QOL (15). Malnourished patients with a low BMI also had a lower QOL. Previous studies have shown that malnutrition is a poor prognostic factor for many cancers (29). Following gastric cancer surgery, particularly after hospital discharge, malnutrition frequently occurs and can intensify (30). In addition, gastrointestinal malabsorption decreases ingestion of food and weight loss, which are not uncommon sequelae after a gastrectomy, and can lead to malnutrition, which in turn leads to prolonged recovery time, decreased physical function, and decreased QOL (31). Patients with gastric cancer who are undernourished and experience significant WL often report decreased QOL, as we observed in this study. Studies have shown that weight loss can affect cancer mortality and chances of cancer recurrence or secondary cancer formation (32). Nutritional interventions can enhance QOL and survival rates of patients with gastric cancer (32, 33).

The essential steps for preventing and managing malnutrition include early detection and tracking of WL, along with suitable nutritional strategies. The QOL of malnourished patients can be influenced by their geographic location and living conditions. HGS is another indicator of subpar QOL in malnourished patients with gastric cancer. Malnourished patients often experience muscle loss and physical decline, resulting in decreased HGS. Higher HGS is associated with a better physical status, as reported previously (34). A risk factor for cancer is sarcopenia because it increases mortality and postoperative complications and reduces treatment response and QOL (35). For cancer survivors, low HGS is connected with poorer QOL. Enhancing muscle strength should be a key focus to improve QOL of those who have survived cancer (36). Consequently, along with providing proper nutritional assistance, the overall treatment plan must include suitable measures for muscle development and rehabilitation to boost the patient’s physical health and overall well-being.

This study had some limitations. Other factors, such as inflammation and body composition, that were not assessed in this study, may impact QOL. We were unable to incorporate these factors into the multivariate analysis as the database we used had limited data on these parameters. Future studies should consider these variables. Secondly, we do not have survival information for the study population, and unfortunately we cannot compare whether the PG-SGA stage assessment or the PG-SGA numerical score analyzed in this study is more beneficial for the survival of patients with gastric cancer. Moreover, both PG-SGA and EORTC QLQC30 questionnaires are asked by professional investigators to study the population, which may cause the deviation of scores due to personal subjective reasons.

Malnutrition, which impacts functional survival and QOL, is common in patients with cancer (37). Malnutrition has negative impacts on the clinical outcome, prolongs hospital stays, and reduces the QOL for patient (38). Our findings may help to focus on certain factors in malnourished gastric cancer patients, thereby improving their QOL.

## Data availability statement

The original contributions presented in the study are included in the article/**Supplementary Material**. Further inquiries can be directed to the corresponding author.

## Ethics statement

The studies involving human participants were reviewed and approved by Medical Ethical Review Committees and Institutional Review Boards of the participating registered hospitals. The patients/participants provided their written informed consent to participate in this study.

## Author contributions

HZ: Conceptualization, Investigation, Methodology, Software, Validation, Writing – original draft, Writing – review & editing. CL: Conceptualization, Methodology, Writing – original draft. GR: Conceptualization, Software, Writing – review & editing. XZ: Formal analysis, Writing – original draft. YC: Formal analysis, Writing – review & editing. SL: Validation, Writing – original draft. XYL: Investigation, Writing – review & editing. JS: Writing – original draft. XGL: Writing – original draft. SL: Writing – review & editing. HS: Funding acquisition, Supervision, Writing – review & editing.
