# Supplementary material for: The quality of life impacting factors in malnourished patients with gastric cancer
Source: Front Oncol. 2024 Apr 25;14:1336859. doi: 10.3389/fonc.2024.1336859 (PMC11079278; doi:10.3389/fonc.2024.1336859)
Supplement: Supplementary file 1 [file DataSheet_1.docx]

Supplementary Figure 1 Procedures for selection of study participants with gastric cancer from the INSCOC study.

The INSCOC study included 5,845 gastric cancer patients with incomplete data.

91 patients with missing BMI data were interpolated.

335 patients with missing WL data were interpolated.

839 patients with missing HGS data were interpolated.

Complete data were obtained from 5845 gastric cancer patients in the INSCOC study.

Supplementary Table 1 Characteristics of patients with gastric cancer.

| **Characteristics** | **Sample size (n=5845)** |
| --- | --- |
| Sex |  |
| Male | 4052 (69.3) |
| Female | 1793 (30.7) |
| Age | 59.00 [51.00, 66.00] |
| TNM stage |  |
| I | 751 (12.8) |
| II | 1166 (19.9) |
| III | 2278 (39.0) |
| IV | 1650 (28.2) |
| BMI | 20.90 [18.76, 23.31] |
| Surgery |  |
| No | 3392 (58.0) |
| Yes | 2453 (42.0) |
| Chemotherapy |  |
| No | 3214 (55.0) |
| Yes | 2631 (45.0) |
| Radiotherapy |  |
| No | 5707 (97.6) |
| Yes | 138 ( 2.4) |
| WL% |  |
| ≤0 | 2496 (42.7) |
| 0～5 | 1561 (26.7) |
| 5～10 | 1212 (20.7) |
| >10 | 576 ( 9.9) |
| HGS |  |
| <18 for women or <28 for men | 2827 (48.4) |
| ≥18 for women or≥28 for men | 3018 (51.6) |
| Education |  |
| Primary education or never attended school | 2120 (36.3) |
| Secondary education | 2928 (50.1) |
| Higher education | 797 (13.6) |
| Occupation |  |
| Mental work | 654 (11.2) |
| Manual work | 2351 (40.2) |
| Retired or other | 2840 (48.6) |
| Residence |  |
| Urban | 2812 (48.1) |
| Rural | 3033 (51.9) |
| PG-SGA |  |
| <4 | 1259 (21.5) |
| ≥4 | 4586 (78.5) |

The summary statistics present N% for categorical variables and median [IQR] deviation for continuous variables.

Supplementary Table 2 Patients self-reported symptoms in PG-SGA on a baseline table with an equal-score score for the global QOL score.

| Symptom | ≥Average golbal QOl score^a^ (n=2765) | <Average golbal QOl score (n=1821) | P |
| --- | --- | --- | --- |
| Have no problem eating |  |  |  |
| No | 1730 (62.6) | 1544 (84.8) | <0.001 |
| Yes | 1035 (37.4) | 277 (15.2) |  |
| Have no appetite |  |  |  |
| No | 2216 (80.1) | 1086 (59.6) | <0.001 |
| Yes | 549 (19.9) | 735 (40.4) |  |
| Nausea |  |  |  |
| No | 2457 (88.9) | 1309 (71.9) | <0.001 |
| Yes | 308 (11.1) | 512 (28.1) |  |
| Vomit |  |  |  |
| No | 2542 (91.9) | 1395 (76.6) | <0.001 |
| Yes | 223 ( 8.1) | 426 (23.4) |  |
| Oral ulcer |  |  |  |
| No | 2746 (99.3) | 1805 (99.1) | 0.578 |
| Yes | 19 ( 0.7) | 16 ( 0.9) |  |
| Constipation |  |  |  |
| No | 2588 (93.6) | 1551 (85.2) | <0.001 |
| Yes | 177 ( 6.4) | 270 (14.8) |  |
| Diarrhea |  |  |  |
| No | 2656 (96.1) | 1721 (94.5) | 0.017 |
| Yes | 109 ( 3.9) | 100 ( 5.5) |  |
| Dry mouth |  |  |  |
| No | 2598 (94.0) | 1619 (88.9) | <0.001 |
| Yes | 167 ( 6.0) | 202 (11.1) |  |
| Tasteless food |  |  |  |
| No | 2650 (95.8) | 1677 (92.1) | <0.001 |
| Yes | 115 ( 4.2) | 144 ( 7.9) |  |
| Food smells bad |  |  |  |
| No | 2709 (98.0) | 1755 (96.4) | 0.001 |
| Yes | 56 ( 2.0) | 66 ( 3.6) |  |
| Dysphagia |  |  |  |
| No | 2613 (94.5) | 1695 (93.1) | 0.056 |
| Yes | 152 ( 5.5) | 126 ( 6.9) |  |
| Early satiety |  |  |  |
| No | 2367 (85.6) | 1527 (83.9) | 0.114 |
| Yes | 398 (14.4) | 294 (16.1) |  |
| Pain |  |  |  |
| No | 2209 (79.9) | 1362 (74.8) | <0.001 |
| Yes | 556 (20.1) | 459 (25.2) |  |

^a^ The Average global QOl score was 82.83, obtained using the EORTC QLQ-C30.The summary statistics present N% for categorical variables.

Supplementary Table 3A Logistic regression analysis was performed to determine the clinical and nutritional parameters associated with the difference in the mean value of global QOL (below the mean value of 83.29) in young adults.

| Characteristics | ≥Global QOL average score (n=1844) | <Global QOL average score (n=1219) | OR | 95%CI | P | OR | 95%CI | P |
| --- | --- | --- | --- | --- | --- | --- | --- | --- |
|  |  |  | Univariate analysis | | | Multivariate analysis | | |
| Sex |  |  |  |  |  |  |  |  |
| Male | 1262 (68.4%) | 716 (58.7%) | 1.00 |  |  |  |  |  |
| Female | 582 (31.6%) | 503 (41.3%) | 1.52 | 1.31-1.77 | <0.001 | 1.53 | 1.29-1.80 | <0.001 |
| Age | 55.00 [48.00, 60.00] | 55.00 [48.00, 60.00] | 1.00 | 0.99-1.01 | 0.590 |  |  |  |
| TNM stage |  |  |  |  |  |  |  |  |
| I | 233 (12.6%) | 138 (11.3%) |  |  |  |  |  |  |
| II | 373 (20.2%) | 216 (17.7%) | 0.98 | 0.75-1.28 | 0.870 | 1.12 | 0.83-1.50 | 0.455 |
| III | 767 (41.6%) | 435 (35.7%) | 0.96 | 0.75-1.22 | 0.725 | 1.13 | 0.86-1.47 | 0.388 |
| IV | 471 (25.5%) | 430 (35.3%) | 1.54 | 1.20-1.97 | <0.001 | 1.93 | 1.45-2.56 | <0.001 |
| BMI | 20.90 [18.95, 23.42] | 19.96 [17.81, 22.42] | 0.91 | 0.89-0.93 | <0.001 | 0.95 | 0.92-0.97 | <0.001 |
| Occupation |  |  |  |  |  |  |  |  |
| Mental work | 270 (14.6%) | 159 (13%) |  |  |  |  |  |  |
| Manual work | 804 (43.6%) | 568 (46.6%) | 1.20 | 0.96-1.50 | 0.110 | 0.97 | 0.71-1.31 | 0.822 |
| Retired or other | 770 (41.8%) | 492 (40.4%) | 1.09 | 0.87-1.36 | 0.480 | 0.94 | 0.71-1.24 | 0.665 |
| Education |  |  |  |  |  |  |  |  |
| Primary education or never attended school | 554 (30%) | 464 (38.1%) |  |  |  |  |  |  |
| Secondary education | 1031 (55.9%) | 593 (48.6%) | 0.69 | 0.59-0.81 | <0.001 | 0.78 | 0.64-0.94 | 0.010 |
| Higher education | 259 (14%) | 162 (13.3%) | 0.75 | 0.59-0.94 | 0.014 | 0.77 | 0.56-1.06 | 0.113 |
| Surgery |  |  |  |  |  |  |  |  |
| No | 1042 (56.5%) | 710 (58.2%) |  |  |  |  |  |  |
| Yes | 802 (43.5%) | 509 (41.8%) | 0.93 | 0.80-1.08 | 0.342 |  |  |  |
| Chemotherapy |  |  |  |  |  |  |  |  |
| No | 958 (52%) | 691 (56.7%) |  |  |  |  |  |  |
| Yes | 886 (48%) | 528 (43.3%) | 0.83 | 0.71-0.96 | 0.010 | 0.89 | 0.74-1.07 | 0.216 |
| Radiotherapy |  |  |  |  |  |  |  |  |
| No | 1809 (98.1%) | 1187 (97.4%) |  |  |  |  |  |  |
| Yes | 35 (1.9%) | 32 (2.6%) | 1.39 | 0.86-2.26 | 0.180 | 1.25 | 0.72-2.14 | 0.426 |
| WL% |  |  |  |  |  |  |  |  |
| ≤0 | 555 (30.1%) | 325 (26.7%) |  |  |  |  |  |  |
| 0～5 | 604 (32.8%) | 333 (27.3%) | 0.94 | 0.78-1.14 | 0.537 | 0.98 | 0.80-1.21 | 0.880 |
| 5～10 | 481 (26.1%) | 375 (30.8%) | 1.33 | 1.10-1.61 | 0.004 | 1.29 | 1.05-1.59 | 0.017 |
| >10 | 204 (11.1%) | 186 (15.3%) | 1.56 | 1.22-1.98 | <0.001 | 1.41 | 1.07-1.84 | 0.013 |
| HGS |  |  |  |  |  |  |  |  |
| <18 for women or <28 for men | 643 (34.9%) | 698 (57.3%) |  |  |  |  |  |  |
| ≥18 for women or≥28 for men | 1201 (65.1%) | 521 (42.7%) | 0.40 | 0.34-0.46 | <0.001 | 0.47 | 0.40-0.56 | <0.001 |
| Residence |  |  |  |  |  |  |  |  |
| Urban | 852 (46.2%) | 529 (43.4%) |  |  |  |  |  |  |
| Rural | 992 (53.8%) | 690 (56.6%) | 1.12 | 0.97-1.30 | 0.126 | 1.03 | 0.86-1.24 | 0.733 |

OR, odds ratio; CI, confidence interval; The summary statistics present N% for categorical variables and median [IQR] deviation for continuous variables.

Supplementary Table 3B Logistic regression analysis was performed to determine the clinical and nutritional parameters associated with the difference between the mean value of global QOL (below the mean value of 81.89) in the elderly.

| Characteristics | ≥Global QOL average score （n=936) | <Global QOL average score （n=587) | OR | 95%CI | P | OR | 95%CI | P |
| --- | --- | --- | --- | --- | --- | --- | --- | --- |
|  |  |  | Univariate analysis | | | Multivariate analysis | | |
| Sex |  |  |  |  |  |  |  |  |
| Male | 751 (80.2%) | 420 (71.6%) |  |  |  |  |  |  |
| Female | 185 (19.8%) | 167 (28.4%) | 1.61 | 1.27-2.05 | <0.001 | 1.64 | 1.26-2.13 | <0.001 |
| Age | 69.00 [67.00, 73.00] | 71.00 [67.00, 75.00] | 1.05 | 1.03-1.07 | <0.001 | 1.03 | 1.01-1.05 | 0.008 |
| TNM stage |  |  |  |  |  |  |  |  |
| I | 121 (12.9%) | 54 (9.2%) |  |  |  |  |  |  |
| II | 178 (19%) | 117 (19.9%) | 1.47 | 0.99-2.19 | 0.056 | 1.60 | 1.04-2.44 | 0.031 |
| III | 380 (40.6%) | 231 (39.4%) | 1.36 | 0.95-1.95 | 0.092 | 1.63 | 1.11-2.41 | 0.013 |
| IV | 257 (27.5%) | 185 (31.5%) | 1.61 | 1.11-2.34 | 0.012 | 2.20 | 1.45-3.33 | <0.001 |
| BMI | 20.83 [18.85, 23.06] | 20.00 [17.93, 22.44] | 0.92 | 0.89-0.95 | <0.001 | 0.94 | 0.91-0.97 | <0.001 |
| Occupation |  |  |  |  |  |  |  |  |
| Mental work | 47 (5%) | 19 (3.2%) |  |  |  |  |  |  |
| Manual work | 316 (33.8%) | 208 (35.4%) | 1.63 | 0.93-2.85 | 0.088 | 1.66 | 0.89-3.12 | 0.114 |
| Retired or other | 573 (61.2%) | 360 (61.3%) | 1.55 | 0.90-2.69 | 0.115 | 1.58 | 0.86-2.88 | 0.138 |
| Education |  |  |  |  |  |  |  |  |
| Primary education or never attended school | 416 (44.4%) | 286 (48.7%) |  |  |  |  |  |  |
| Secondary education | 407 (43.5%) | 228 (38.8%) | 0.81 | 0.65-1.02 | 0.070 | 0.91 | 0.70-1.18 | 0.484 |
| Higher education | 113 (12.1%) | 73 (12.4%) | 0.94 | 0.68-1.31 | 0.712 | 1.01 | 0.68-1.50 | 0.953 |
| Surgery |  |  |  |  |  |  |  |  |
| No | 535 (57.2%) | 352 (60%) |  |  |  |  |  |  |
| Yes | 401 (42.8%) | 235 (40%) | 0.89 | 0.72-1.10 | 0.280 |  |  |  |
| Chemotherapy |  |  |  |  |  |  |  |  |
| No | 505 (54%) | 400 (68.1%) |  |  |  |  |  |  |
| Yes | 431 (46%) | 187 (31.9%) | 0.55 | 0.44-0.68 | <0.001 | 0.57 | 0.44-0.74 | <0.001 |
| Radiotherapy |  |  |  |  |  |  |  |  |
| No | 908 (97%) | 573 (97.6%) |  |  |  |  |  |  |
| Yes | 28 (3%) | 14 (2.4%) | 0.79 | 0.41-1.52 | 0.483 |  |  |  |
| WL% |  |  |  |  |  |  |  |  |
| ≤0 | 363 (38.8%) | 175 (29.8%) |  |  |  |  |  |  |
| 0～5 | 270 (28.8%) | 176 (30%) | 1.35 | 1.04-1.76 | 0.024 | 1.37 | 1.04-1.82 | 0.027 |
| 5～10 | 209 (22.3%) | 146 (24.9%) | 1.45 | 1.10-1.91 | 0.009 | 1.43 | 1.06-1.93 | 0.018 |
| >10 | 94 (10%) | 90 (15.3%) | 1.99 | 1.41-2.79 | <0.001 | 1.84 | 1.26-2.67 | 0.001 |
| HGS |  |  |  |  |  |  |  |  |
| <18 for women or <28 for men | 574 (61.3%) | 443 (75.5%) |  |  |  |  |  |  |
| ≥18 for women or≥28 for men | 362 (38.7%) | 144 (24.5%) | 0.52 | 0.41-0.65 | <0.001 | 0.61 | 0.47-0.78 | <0.001 |
| Residence |  |  |  |  |  |  |  |  |
| Urban | 492 (52.6%) | 315 (53.7%) |  |  |  |  |  |  |
| Rural | 444 (47.4%) | 272 (46.3%) | 0.96 | 0.78-1.18 | 0.676 |  |  |  |

OR, odds ratio; CI, confidence interval; The summary statistics present N% for categorical variables and median [IQR] deviation for continuous variables.

Supplementary Table 4A Logistic regression analysis was performed to determine the clinical and nutritional parameters associated with the difference in the mean value of physical function (below the mean value of 80.39) in young adults.

| Characteristics | ≥Physical function average score (n=1789) | <Physical function average score (n=1274) | OR | 95%CI | P | OR | 95%CI | P |
| --- | --- | --- | --- | --- | --- | --- | --- | --- |
|  |  |  | Univariate analysis | | | Multivariate analysis | | |
| Sex |  |  |  |  |  |  |  |  |
| Male | 1206 (67.4%) | 772 (60.6%) |  |  |  |  |  |  |
| Female | 583 (32.6%) | 502 (39.4%) | 1.35 | 1.16-1.56 | <0.001 | 1.33 | 1.12-1.57 | 0.001 |
| Age | 54.00 [48.00, 60.00] | 55.50 [49.00, 60.00] | 1.01 | 1.00-1.02 | 0.017 | 1.01 | 1.00-1.02 | 0.165 |
| TNM stage |  |  |  |  |  |  |  |  |
| I | 225 (12.6%) | 146 (11.5%) |  |  |  |  |  |  |
| II | 360 (20.1%) | 229 (18%) | 0.98 | 0.75-1.28 | 0.884 | 1.03 | 0.77-1.39 | 0.830 |
| III | 760 (42.5%) | 442 (34.7%) | 0.9 | 0.71-1.14 | 0.369 | 0.86 | 0.66-1.12 | 0.270 |
| IV | 444 (24.8%) | 457 (35.9%) | 1.59 | 1.24-2.03 | <0.001 | 1.53 | 1.15-2.04 | 0.004 |
| BMI | 21.01 [18.95, 23.42] | 19.88 [17.92, 22.31] | 0.9 | 0.88-0.93 | <0.001 | 0.95 | 0.92-0.97 | <0.001 |
| Occupation |  |  |  |  |  |  |  |  |
| Mental work | 259 (14.5%) | 170 (13.3%) |  |  |  |  |  |  |
| Manual work | 807 (45.1%) | 565 (44.3%) | 1.07 | 0.85-1.33 | 0.568 |  |  |  |
| Retired or other | 723 (40.4%) | 539 (42.3%) | 1.14 | 0.91-1.42 | 0.264 |  |  |  |
| Education |  |  |  |  |  |  |  |  |
| Primary education or never attended school | 535 (29.9%) | 483 (37.9%) |  |  |  |  |  |  |
| Secondary education | 1014 (56.7%) | 610 (47.9%) | 0.67 | 0.57-0.78 | <0.001 | 0.71 | 0.59-0.85 | <0.001 |
| Higher education | 240 (13.4%) | 181 (14.2%) | 0.84 | 0.66-1.05 | 0.123 | 0.83 | 0.64-1.08 | 0.172 |
| Surgery |  |  |  |  |  |  |  |  |
| No | 957 (53.5%) | 795 (62.4%) |  |  |  |  |  |  |
| Yes | 832 (46.5%) | 479 (37.6%) | 0.69 | 0.60-0.80 | <0.001 | 0.75 | 0.62-0.92 | 0.005 |
| Chemotherapy |  |  |  |  |  |  |  |  |
| No | 970 (54.2%) | 679 (53.3%) |  |  |  |  |  |  |
| Yes | 819 (45.8%) | 595 (46.7%) | 1.04 | 0.90-1.20 | 0.613 |  |  |  |
| Radiotherapy |  |  |  |  |  |  |  |  |
| No | 1749 (97.8%) | 1247 (97.9%) |  |  |  |  |  |  |
| Yes | 40 (2.2%) | 27 (2.1%) | 0.95 | 0.58-1.55 | 0.828 |  |  |  |
| WL% |  |  |  |  |  |  |  |  |
| ≤0 | 537 (30%) | 343 (26.9%) |  |  |  |  |  |  |
| 0～5 | 588 (32.9%) | 349 (27.4%) | 0.93 | 0.77-1.12 | 0.448 | 0.98 | 0.79-1.20 | 0.821 |
| 5～10 | 469 (26.2%) | 387 (30.4%) | 1.29 | 1.07-1.56 | 0.009 | 1.20 | 0.97-1.48 | 0.088 |
| >10 | 195 (10.9%) | 195 (15.3%) | 1.57 | 1.23-1.99 | <0.001 | 1.30 | 1.00-1.70 | 0.054 |
| HGS |  |  |  |  |  |  |  |  |
| <18 for women or <28 for men | 594 (33.2%) | 747 (58.6%) |  |  |  |  |  |  |
| ≥18 for women or≥28 for men | 1195 (66.8%) | 527 (41.4%) | 0.35 | 0.30-0.41 | <0.001 | 0.43 | 0.36-0.51 | <0.001 |
| Residence |  |  |  |  |  |  |  |  |
| Urban | 790 (44.2%) | 591 (46.4%) |  |  |  |  |  |  |
| Rural | 999 (55.8%) | 683 (53.6%) | 0.91 | 0.79-1.06 | 0.221 |  |  |  |

OR, odds ratio; CI, confidence interval; The summary statistics present N% for categorical variables and median [IQR] deviation for continuous variables.

Supplementary Table 4B Logistic regression analysis was performed to determine the clinical and nutritional parameters associated with the difference between the mean value of physical function (below the mean value of 75.21) in the elderly.

| Characteristics | ≥Physical function average score (n=936) | <Physical function average score (n=587) | OR | OR(95%CI) | P | OR | OR(95%CI) | P |
| --- | --- | --- | --- | --- | --- | --- | --- | --- |
|  |  |  | Univariate analysis | | | Multivariate analysis | | |
| Sex |  |  |  |  |  |  |  |  |
| Male | 756 (80.8%) | 415 (70.7%) |  |  |  |  |  |  |
| Female | 180 (19.2%) | 172 (29.3%) | 1.74 | 1.37-2.21 | <0.001 | 1.90 | 1.45-2.50 | <0.001 |
| Age | 69.00 [66.00, 73.00] | 71.00 [67.00, 76.00] | 1.07 | 1.05-1.10 | <0.001 | 1.06 | 1.04-1.09 | <0.001 |
| TNM stage |  |  |  |  |  |  |  |  |
| I | 131 (14%) | 44 (7.5%) |  |  |  |  |  |  |
| II | 176 (18.8%) | 119 (20.3%) | 2.01 | 1.33-3.04 | <0.001 | 2.01 | 1.27-3.16 | 0.003 |
| III | 386 (41.2%) | 225 (38.3%) | 1.74 | 1.19-2.54 | 0.004 | 1.89 | 1.24-2.87 | 0.003 |
| IV | 243 (26%) | 199 (33.9%) | 2.44 | 1.65-3.60 | <0.001 | 2.53 | 1.61-3.96 | <0.001 |
| BMI | 20.96 [18.82, 23.15] | 19.91 [17.75, 22.32] | 0.90 | 0.87-0.93 | <0.001 | 0.94 | 0.90-0.97 | <0.001 |
| Occupation |  |  |  |  |  |  |  |  |
| Mental work | 47 (5%) | 19 (3.2%) |  |  |  |  |  |  |
| Manual work | 326 (34.8%) | 198 (33.7%) | 1.50 | 0.86-2.63 | 0.155 | 1.77 | 0.90-3.50 | 0.100 |
| Retired or other | 563 (60.1%) | 370 (63%) | 1.63 | 0.94-2.81 | 0.083 | 1.48 | 0.78-2.81 | 0.228 |
| Education |  |  |  |  |  |  |  |  |
| Primary education or never attended school | 426 (45.5%) | 276 (47%) |  |  |  |  |  |  |
| Secondary education | 409 (43.7%) | 226 (38.5%) | 0.85 | 0.68-1.06 | 0.160 | 0.90 | 0.69-1.19 | 0.459 |
| Higher education | 101 (10.8%) | 85 (14.5%) | 1.30 | 0.94-1.80 | 0.116 | 1.21 | 0.80-1.82 | 0.359 |
| Surgery |  |  |  |  |  |  |  |  |
| No | 510 (54.5%) | 377 (64.2%) |  |  |  |  |  |  |
| Yes | 426 (45.5%) | 210 (35.8%) | 0.67 | 0.54-0.82 | <0.001 | 0.42 | 0.30-0.60 | <0.001 |
| Chemotherapy |  |  |  |  |  |  |  |  |
| No | 526 (56.2%) | 379 (64.6%) |  |  |  |  |  |  |
| Yes | 410 (43.8%) | 208 (35.4%) | 0.70 | 0.57-0.87 | 0.001 | 0.47 | 0.34-0.65 | <0.001 |
| Radiotherapy |  |  |  |  |  |  |  |  |
| No | 910 (97.2%) | 571 (97.3%) |  |  |  |  |  |  |
| Yes | 26 (2.8%) | 16 (2.7%) | 0.98 | 0.52-1.84 | 0.952 |  |  |  |
| WL% |  |  |  |  |  |  |  |  |
| ≤0 | 356 (38%) | 182 (31%) |  |  |  |  |  |  |
| 0～5 | 272 (29.1%) | 174 (29.6%) | 1.25 | 0.96-1.62 | 0.092 | 1.32 | 0.99-1.76 | 0.063 |
| 5～10 | 217 (23.2%) | 138 (23.5%) | 1.24 | 0.94-1.64 | 0.124 | 1.24 | 0.91-1.70 | 0.169 |
| >10 | 91 (9.7%) | 93 (15.8%) | 2.00 | 1.42-2.81 | <0.001 | 1.72 | 1.18-2.52 | 0.005 |
| HGS |  |  |  |  |  |  |  |  |
| <18 for women or <28 for men | 555 (59.3%) | 462 (78.7%) |  |  |  |  |  |  |
| ≥18 for women or≥28 for men | 381 (40.7%) | 125 (21.3%) | 0.39 | 0.31-0.50 | <0.001 | 0.48 | 0.37-0.62 | <0.001 |
| Residence |  |  |  |  |  |  |  |  |
| Urban | 471 (50.3%) | 336 (57.2%) |  |  |  |  |  |  |
| Rural | 465 (49.7%) | 251 (42.8%) | 0.76 | 0.61-0.93 | 0.009 | 0.85 | 0.65-1.13 | 0.264 |

OR, odds ratio; CI, confidence interval; The summary statistics present N% for categorical variables and median [IQR] deviation for continuous variables.

Supplementary Table 5A Logistic regression analysis was used to identify clinical and nutritional parameters associated with higher than average fatigue (23.29) in young adults.

| Characteristics | <Fatigue function average score (n=1793) | ≥Fatigue average score (n=1270) | OR | 95%CI | P | OR | 95%CI | P |
| --- | --- | --- | --- | --- | --- | --- | --- | --- |
|  |  |  | Univariate analysis | | | Multivariate analysis | | |
| Sex |  |  |  |  |  |  |  |  |
| Male | 1203 (67.1%) | 775 (61%) |  |  |  |  |  |  |
| Female | 590 (32.9%) | 495 (39%) | 1.30 | 1.12-1.51 | <0.001 | 1.27 | 1.08-1.49 | 0.004 |
| Age | 55.00 [48.00, 60.00] | 55.00 [48.00, 60.00] | 1.00 | 1.00-1.01 | 0.286 |  |  |  |
| TNM stage |  |  |  |  |  |  |  |  |
| I | 240 (13.4%) | 131 (10.3%) |  |  |  |  |  |  |
| II | 370 (20.6%) | 219 (17.2%) | 1.08 | 0.83-1.42 | 0.557 | 1.11 | 0.83-1.48 | 0.499 |
| III | 727 (40.5%) | 475 (37.4%) | 1.20 | 0.94-1.53 | 0.146 | 1.18 | 0.90-1.53 | 0.225 |
| IV | 456 (25.4%) | 445 (35%) | 1.79 | 1.39-2.30 | <0.001 | 1.64 | 1.23-2.18 | <0.001 |
| BMI | 20.96 [18.95, 23.53] | 19.86 [17.86, 22.31] | 0.90 | 0.88-0.92 | <0.001 | 0.94 | 0.92-0.97 | <0.001 |
| Occupation |  |  |  |  |  |  |  |  |
| Mental work | 249 (13.9%) | 180 (14.2%) |  |  |  |  |  |  |
| Manual work | 797 (44.5%) | 575 (45.3%) | 1.00 | 0.80-1.24 | 0.986 |  |  |  |
| Retired or other | 747 (41.7%) | 515 (40.6%) | 0.95 | 0.76-1.19 | 0.676 |  |  |  |
| Education |  |  |  |  |  |  |  |  |
| Primary education or never attended school | 569 (31.7%) | 449 (35.4%) |  |  |  |  |  |  |
| Secondary education | 982 (54.8%) | 642 (50.6%) | 0.83 | 0.71-0.97 | 0.020 | 0.93 | 0.78-1.11 | 0.434 |
| Higher education | 242 (13.5%) | 179 (14.1%) | 0.94 | 0.75-1.18 | 0.581 | 0.97 | 0.75-1.24 | 0.785 |
| Surgery |  |  |  |  |  |  |  |  |
| No | 969 (54%) | 783 (61.7%) |  |  |  |  |  |  |
| Yes | 824 (46%) | 487 (38.3%) | 0.73 | 0.63-0.85 | <0.001 | 0.76 | 0.62-0.92 | 0.006 |
| Chemotherapy |  |  |  |  |  |  |  |  |
| No | 975 (54.4%) | 674 (53.1%) |  |  |  |  |  |  |
| Yes | 818 (45.6%) | 596 (46.9%) | 1.05 | 0.91-1.22 | 0.475 |  |  |  |
| Radiotherapy |  |  |  |  |  |  |  |  |
| No | 1760 (98.2%) | 1236 (97.3%) |  |  |  |  |  |  |
| Yes | 33 (1.8%) | 34 (2.7%) | 1.47 | 0.90-2.38 | 0.121 | 1.06 | 0.62-1.80 | 0.843 |
| WL% |  |  |  |  |  |  |  |  |
| ≤0 | 549 (30.6%) | 331 (26.1%) |  |  |  |  |  |  |
| 0～5 | 585 (32.6%) | 352 (27.7%) | 1.00 | 0.83-1.21 | 0.984 | 1.06 | 0.87-1.30 | 0.549 |
| 5～10 | 478 (26.7%) | 378 (29.8%) | 1.31 | 1.08-1.59 | 0.006 | 1.27 | 1.04-1.56 | 0.021 |
| >10 | 181 (10.1%) | 209 (16.5%) | 1.92 | 1.50-2.44 | <0.001 | 1.68 | 1.29-2.18 | <0.001 |
| HGS |  |  |  |  |  |  |  |  |
| <18 for women or <28 for men | 623 (34.7%) | 718 (56.5%) |  |  |  |  |  |  |
| ≥18 for women or≥28 for men | 1170 (65.3%) | 552 (43.5%) | 0.41 | 0.35-0.47 | <0.001 | 0.49 | 0.42-0.57 | <0.001 |
| Residence |  |  |  |  |  |  |  |  |
| Urban | 803 (44.8%) | 578 (45.5%) |  |  |  |  |  |  |
| Rural | 990 (55.2%) | 692 (54.5%) | 0.97 | 0.84-1.12 | 0.691 |  |  |  |

OR, odds ratio; CI, confidence interval; The summary statistics present N% for categorical variables and median [IQR] deviation for continuous variables.

Supplementary Table 5B Logistic regression analysis was used to determine the clinical and nutritional parameters associated with higher than average fatigue (25.87) in older adults.

| Characteristics | <Fatigue function average score (n=846) | ≥Fatigue average score (n=677) | OR | 95%CI | P | OR | 95%CI | P |
| --- | --- | --- | --- | --- | --- | --- | --- | --- |
|  |  |  | Univariate analysis | | | Multivariate analysis | | |
| Sex |  |  |  |  |  |  |  |  |
| Male | 681 (80.5%) | 490 (72.4%) |  |  |  |  |  |  |
| Female | 165 (19.5%) | 187 (27.6%) | 1.58 | 1.24-2.00 | <0.001 | 1.63 | 1.26-2.11 | <0.001 |
| Age | 69.00 [67.00, 73.00] | 71.00 [67.00, 75.00] | 1.04 | 1.02-1.06 | <0.001 | 1.03 | 1.01-1.05 | 0.007 |
| TNM stage |  |  |  |  |  |  |  |  |
| I | 105 (12.4%) | 70 (10.3%) |  |  |  |  |  |  |
| II | 148 (17.5%) | 147 (21.7%) | 1.49 | 1.02-2.18 | 0.039 | 1.39 | 0.93-2.09 | 0.107 |
| III | 355 (42%) | 256 (37.8%) | 1.08 | 0.77-1.52 | 0.653 | 1.05 | 0.72-1.51 | 0.808 |
| IV | 238 (28.1%) | 204 (30.1%) | 1.29 | 0.90-1.83 | 0.166 | 1.15 | 0.77-1.72 | 0.488 |
| BMI | 20.83 [18.76, 23.14] | 20.20 [17.96, 22.48] | 0.93 | 0.90-0.96 | <0.001 | 0.96 | 0.93-0.99 | 0.021 |
| Occupation |  |  |  |  |  |  |  |  |
| Mental work | 37 (4.4%) | 29 (4.3%) |  |  |  |  |  |  |
| Manual work | 294 (34.8%) | 230 (34%) | 1 | 0.60-1.67 | 0.994 |  |  |  |
| Retired or other | 515 (60.9%) | 418 (61.7%) | 1.04 | 0.63-1.71 | 0.892 |  |  |  |
| Education |  |  |  |  |  |  |  |  |
| Primary education or never attended school | 380 (44.9%) | 322 (47.6%) |  |  |  |  |  |  |
| Secondary education | 370 (43.7%) | 265 (39.1%) | 0.85 | 0.68-1.05 | 0.128 | 0.89 | 0.70-1.14 | 0.353 |
| Higher education | 96 (11.3%) | 90 (13.3%) | 1.11 | 0.80-1.53 | 0.54 | 1.09 | 0.77-1.56 | 0.62 |
| Surgery |  |  |  |  |  |  |  |  |
| No | 472 (55.8%) | 415 (61.3%) |  |  |  |  |  |  |
| Yes | 374 (44.2%) | 262 (38.7%) | 0.8 | 0.65-0.98 | 0.03 | 0.5 | 0.36-0.69 | <0.001 |
| Chemotherapy |  |  |  |  |  |  |  |  |
| No | 479 (56.6%) | 426 (62.9%) |  |  |  |  |  |  |
| Yes | 367 (43.4%) | 251 (37.1%) | 0.77 | 0.63-0.95 | 0.013 | 0.64 | 0.47-0.86 | 0.003 |
| Radiotherapy |  |  |  |  |  |  |  |  |
| No | 820 (96.9%) | 661 (97.6%) |  |  |  |  |  |  |
| Yes | 26 (3.1%) | 16 (2.4%) | 0.76 | 0.41-1.43 | 0.402 |  |  |  |
| WL% |  |  |  |  |  |  |  |  |
| ≤0 | 328 (38.8%) | 210 (31%) |  |  |  |  |  |  |
| 0～5 | 245 (29%) | 201 (29.7%) | 1.28 | 0.99-1.65 | 0.056 | 1.3 | 0.99-1.71 | 0.054 |
| 5～10 | 187 (22.1%) | 168 (24.8%) | 1.4 | 1.07-1.84 | 0.014 | 1.43 | 1.07-1.92 | 0.014 |
| >10 | 86 (10.2%) | 98 (14.5%) | 1.78 | 1.27-2.49 | <0.001 | 1.68 | 1.17-2.41 | 0.005 |
| HGS |  |  |  |  |  |  |  |  |
| <18 for women or <28 for men | 515 (60.9%) | 502 (74.2%) |  |  |  |  |  |  |
| ≥18 for women or≥28 for men | 331 (39.1%) | 175 (25.8%) | 0.54 | 0.44-0.68 | <0.001 | 0.64 | 0.51-0.81 | <0.001 |
| Residence |  |  |  |  |  |  |  |  |
| Urban | 439 (51.9%) | 368 (54.4%) |  |  |  |  |  |  |
| Rural | 407 (48.1%) | 309 (45.6%) | 0.91 | 0.74-1.11 | 0.338 |  |  |  |

OR, odds ratio; CI, confidence interval; The summary statistics present N% for categorical variables and median [IQR] deviation for continuous variables.

Supplementary Table 6A Logistic regression analysis was used to identify clinical and nutritional parameters associated with higher than average appetite loss (20.53) in young adults.

| Characteristics | <Appetite loss average score (n=1689) | ≥Appetite loss average score (n=1374) | OR | 95%CI | P | OR | 95%CI | P |
| --- | --- | --- | --- | --- | --- | --- | --- | --- |
|  |  |  | Univariate analysis | | | Multivariate analysis | | |
| Sex |  |  |  |  |  |  |  |  |
| Male | 1158 (68.6%) | 820 (59.7%) |  |  |  |  |  |  |
| Female | 531 (31.4%) | 554 (40.3%) | 1.47 | 1.27-1.71 | <0.001 | 1.43 | 1.22-1.68 | <0.001 |
| Age | 55.00 [48.00, 60.00] | 55.00 [48.00, 60.00] | 1.00 | 1.00-1.01 | 0.420 |  |  |  |
| TNM stage |  |  |  |  |  |  |  |  |
| I | 217 (12.8%) | 154 (11.2%) |  |  |  |  |  |  |
| II | 339 (20.1%) | 250 (18.2%) | 1.04 | 0.80-1.35 | 0.775 | 1.11 | 0.84-1.47 | 0.463 |
| III | 708 (41.9%) | 494 (36%) | 0.98 | 0.78-1.25 | 0.888 | 1.10 | 0.85-1.42 | 0.467 |
| IV | 425 (25.2%) | 476 (34.6%) | 1.58 | 1.24-2.02 | <0.001 | 1.72 | 1.30-2.26 | <0.001 |
| BMI | 20.81 [18.83, 23.39] | 20.20 [18.08, 22.49] | 0.93 | 0.91-0.95 | <0.001 | 0.96 | 0.94-0.99 | 0.002 |
| Occupation |  |  |  |  |  |  |  |  |
| Mental work | 241 (14.3%) | 188 (13.7%) |  |  |  |  |  |  |
| Manual work | 723 (42.8%) | 649 (47.2%) | 1.15 | 0.93-1.43 | 0.207 |  |  |  |
| Retired or other | 725 (42.9%) | 537 (39.1%) | 0.95 | 0.76-1.18 | 0.646 |  |  |  |
| Education |  |  |  |  |  |  |  |  |
| Primary education or never attended school | 511 (30.3%) | 507 (36.9%) |  |  |  |  |  |  |
| Secondary education | 943 (55.8%) | 681 (49.6%) | 0.73 | 0.62-0.85 | <0.001 | 0.82 | 0.69-0.98 | 0.028 |
| Higher education | 235 (13.9%) | 186 (13.5%) | 0.80 | 0.63-1.00 | 0.052 | 0.82 | 0.63-1.06 | 0.136 |
| Surgery |  |  |  |  |  |  |  |  |
| No | 948 (56.1%) | 804 (58.5%) |  |  |  |  |  |  |
| Yes | 741 (43.9%) | 570 (41.5%) | 0.91 | 0.79-1.05 | 0.184 | 0.76 | 0.59-0.99 | 0.039 |
| Chemotherapy |  |  |  |  |  |  |  |  |
| No | 890 (52.7%) | 759 (55.2%) |  |  |  |  |  |  |
| Yes | 799 (47.3%) | 615 (44.8%) | 0.90 | 0.78-1.04 | 0.160 | 0.80 | 0.63-1.01 | 0.061 |
| Radiotherapy |  |  |  |  |  |  |  |  |
| No | 1654 (97.9%) | 1342 (97.7%) |  |  |  |  |  |  |
| Yes | 35 (2.1%) | 32 (2.3%) | 1.13 | 0.69-1.83 | 0.629 |  |  |  |
| WL% |  |  |  |  |  |  |  |  |
| ≤0 | 511 (30.3%) | 369 (26.9%) |  |  |  |  |  |  |
| 0～5 | 517 (30.6%) | 420 (30.6%) | 1.13 | 0.93-1.35 | 0.214 | 1.22 | 1.00-1.48 | 0.052 |
| 5～10 | 467 (27.6%) | 389 (28.3%) | 1.15 | 0.95-1.39 | 0.140 | 1.18 | 0.96-1.44 | 0.110 |
| >10 | 194 (11.5%) | 196 (14.3%) | 1.40 | 1.10-1.78 | 0.006 | 1.41 | 1.08-1.83 | 0.010 |
| HGS |  |  |  |  |  |  |  |  |
| <18 for women or <28 for men | 659 (39%) | 682 (49.6%) |  |  |  |  |  |  |
| ≥18 for women or≥28 for men | 1030 (61%) | 692 (50.4%) | 0.65 | 0.56-0.75 | <0.001 | 0.77 | 0.66-0.90 | 0.001 |
| Residence |  |  |  |  |  |  |  |  |
| Urban | 787 (46.6%) | 594 (43.2%) |  |  |  |  |  |  |
| Rural | 902 (53.4%) | 780 (56.8%) | 1.15 | 0.99-1.32 | 0.063 | 1.04 | 0.88-1.23 | 0.649 |

OR, odds ratio; CI, confidence interval; The summary statistics present N% for categorical variables and median [IQR] deviation for continuous variables.

Supplementary Table 6B Logistic regression analysis was used to determine the clinical and nutritional parameters associated with higher than average appetite loss (23.64) in older adults.

| Characteristics | <Appetite loss average score (n=777) | ≥Appetite loss average score (n=746) | OR | 95%CI | P | OR | 95%CI | P |
| --- | --- | --- | --- | --- | --- | --- | --- | --- |
|  |  |  | Univariate analysis | | | Multivariate analysis | | |
| Sex |  |  |  |  |  |  |  |  |
| Male | 628 (80.8%) | 543 (72.8%) |  |  |  |  |  |  |
| Female | 149 (19.2%) | 203 (27.2%) | 1.58 | 1.24-2.00 | <0.001 | 1.65 | 1.29-2.13 | <0.001 |
| Age | 70.00 [67.00, 74.00] | 70.00 [67.00, 75.00] | 1.02 | 1.00-1.04 | 0.039 | 1.01 | 0.99-1.04 | 0.176 |
| TNM stage |  |  |  |  |  |  |  |  |
| I | 94 (12.1%) | 81 (10.9%) |  |  |  |  |  |  |
| II | 155 (19.9%) | 140 (18.8%) | 1.05 | 0.72-1.52 | 0.806 | 1.04 | 0.70-1.54 | 0.848 |
| III | 321 (41.3%) | 290 (38.9%) | 1.05 | 0.75-1.47 | 0.783 | 1.05 | 0.74-1.51 | 0.781 |
| IV | 207 (26.6%) | 235 (31.5%) | 1.32 | 0.93-1.87 | 0.124 | 1.22 | 0.82-1.80 | 0.321 |
| BMI | 20.68 [18.59, 23.05] | 20.43 [18.09, 22.66] | 0.96 | 0.93-0.99 | 0.014 | 0.98 | 0.95-1.01 | 0.191 |
| Occupation |  |  |  |  |  |  |  |  |
| Mental work | 35 (4.5%) | 31 (4.2%) |  |  |  |  |  |  |
| Manual work | 260 (33.5%) | 264 (35.4%) | 1.15 | 0.69-1.91 | 0.602 |  |  |  |
| Retired or other | 482 (62%) | 451 (60.5%) | 1.06 | 0.64-1.74 | 0.83 |  |  |  |
| Education |  |  |  |  |  |  |  |  |
| Primary education or never attended school | 355 (45.7%) | 347 (46.5%) |  |  |  |  |  |  |
| Secondary education | 332 (42.7%) | 303 (40.6%) | 0.93 | 0.75-1.16 | 0.531 |  |  |  |
| Higher education | 90 (11.6%) | 96 (12.9%) | 1.09 | 0.79-1.51 | 0.597 |  |  |  |
| Surgery |  |  |  |  |  |  |  |  |
| No | 421 (54.2%) | 466 (62.5%) |  |  |  |  |  |  |
| Yes | 356 (45.8%) | 280 (37.5%) | 0.71 | 0.58-0.87 | 0.001 | 0.67 | 0.52-0.87 | 0.003 |
| Chemotherapy |  |  |  |  |  |  |  |  |
| No | 461 (59.3%) | 444 (59.5%) |  |  |  |  |  |  |
| Yes | 316 (40.7%) | 302 (40.5%) | 0.99 | 0.81-1.22 | 0.941 |  |  |  |
| Radiotherapy |  |  |  |  |  |  |  |  |
| No | 763 (98.2%) | 718 (96.2%) |  |  |  |  |  |  |
| Yes | 14 (1.8%) | 28 (3.8%) | 2.13 | 1.11-4.07 | 0.023 | 1.83 | 0.92-3.64 | 0.087 |
| WL% |  |  |  |  |  |  |  |  |
| ≤0 | 308 (39.6%) | 230 (30.8%) |  |  |  |  |  |  |
| 0～5 | 211 (27.2%) | 235 (31.5%) | 1.49 | 1.16-1.92 | 0.002 | 1.55 | 1.19-2.03 | 0.001 |
| 5～10 | 170 (21.9%) | 185 (24.8%) | 1.46 | 1.11-1.91 | 0.006 | 1.6 | 1.21-2.13 | 0.001 |
| >10 | 88 (11.3%) | 96 (12.9%) | 1.46 | 1.04-2.04 | 0.027 | 1.48 | 1.04-2.12 | 0.031 |
| HGS |  |  |  |  |  |  |  |  |
| <18 for women or <28 for men | 482 (62%) | 535 (71.7%) |  |  |  |  |  |  |
| ≥18 for women or≥28 for men | 295 (38%) | 211 (28.3%) | 0.64 | 0.52-0.80 | <0.001 | 0.69 | 0.54-0.86 | 0.001 |
| Residence |  |  |  |  |  |  |  |  |
| Urban | 406 (52.3%) | 401 (53.8%) |  |  |  |  |  |  |
| Rural | 371 (47.7%) | 345 (46.2%) | 0.94 | 0.77-1.15 | 0.557 |  |  |  |

OR, odds ratio; CI, confidence interval; The summary statistics present N% for categorical variables and median [IQR] deviation for continuous variables.

Supplementary Table 7 Univariate logistic regression between patients' self-reported PG-SGA symptoms and poor global QOL average score (<82.83) in the past 2 weeks.

| Symptom | ≥Average global QOl score (n=2765) | <Average global QOl score (n=1821) | OR | 95%CI | P-Value |
| --- | --- | --- | --- | --- | --- |
| Have no problem eating |  |  |  |  |  |
| No | 1730 (62.6%) | 1544 (84.8%) |  |  |  |
| Yes | 1035 (37.4%) | 277 (15.2%) | 0.30 | 0.26-0.35 | <0.001 |
| Have no appetite |  |  |  |  |  |
| No | 2216 (80.1%) | 1086 (59.6%) |  |  |  |
| Yes | 549 (19.9%) | 735 (40.4%) | 2.73 | 2.39-3.12 | <0.001 |
| Nausea |  |  |  |  |  |
| No | 2457 (88.9%) | 1309 (71.9%) |  |  |  |
| Yes | 308 (11.1%) | 512 (28.1%) | 3.12 | 2.67-3.65 | <0.001 |
| Vomit |  |  |  |  |  |
| No | 2542 (91.9%) | 1395 (76.6%) |  |  |  |
| Yes | 223 (8.1%) | 426 (23.4%) | 3.48 | 2.92-4.15 | <0.001 |
| Oral ulcer |  |  |  |  |  |
| No | 2746 (99.3%) | 1805 (99.1%) |  |  |  |
| Yes | 19 (0.7%) | 16 (0.9%) | 1.28 | 0.66-2.50 | 0.467 |
| Constipation |  |  |  |  |  |
| No | 2588 (93.6%) | 1551 (85.2%) |  |  |  |
| Yes | 177 (6.4%) | 270 (14.8%) | 2.55 | 2.08-3.11 | <0.001 |
| Diarrhea |  |  |  |  |  |
| No | 2656 (96.1%) | 1721 (94.5%) |  |  |  |
| Yes | 109 (3.9%) | 100 (5.5%) | 1.42 | 1.07-1.87 | 0.014 |
| Dry mouth |  |  |  |  |  |
| No | 2598 (94%) | 1619 (88.9%) |  |  |  |
| Yes | 167 (6%) | 202 (11.1%) | 1.94 | 1.57-2.40 | <0.001 |
| Tasteless food |  |  |  |  |  |
| No | 2650 (95.8%) | 1677 (92.1%) |  |  |  |
| Yes | 115 (4.2%) | 144 (7.9%) | 1.98 | 1.54-2.55 | <0.001 |
| Food smells bad |  |  |  |  |  |
| No | 2709 (98%) | 1755 (96.4%) |  |  |  |
| Yes | 56 (2%) | 66 (3.6%) | 1.82 | 1.27-2.61 | 0.001 |
| Dysphagia |  |  |  |  |  |
| No | 2613 (94.5%) | 1695 (93.1%) |  |  |  |
| Yes | 152 (5.5%) | 126 (6.9%) | 1.28 | 1.00-1.63 | 0.049 |
| Early satiety |  |  |  |  |  |
| No | 2367 (85.6%) | 1527 (83.9%) |  |  |  |
| Yes | 398 (14.4%) | 294 (16.1%) | 1.15 | 0.97-1.35 | 0.105 |
| Pain |  |  |  |  |  |
| No | 2209 (79.9%) | 1362 (74.8%) |  |  |  |
| Yes | 556 (20.1%) | 459 (25.2%) | 1.34 | 1.16-1.54 | <0.001 |

OR, odds ratio; CI, confidence interval; The summary statistics present N% for categorical variables and median [IQR] deviation for continuous variables.
